# Heterogeneity of Potassium Channels in Human Embryonic Stem Cell-Derived Retinal Pigment Epithelium

**DOI:** 10.1093/stcltm/szac029

**Published:** 2022-05-27

**Authors:** Iina Korkka, Heli Skottman, Soile Nymark

**Affiliations:** BioMediTech, Faculty of Medicine and Health Technology, Tampere University, Tampere, Finland; BioMediTech, Faculty of Medicine and Health Technology, Tampere University, Tampere, Finland; BioMediTech, Faculty of Medicine and Health Technology, Tampere University, Tampere, Finland

**Keywords:** retinal pigment epithelium (RPE), K^+^ ion channels, embryonic stem cells, pluripotent stem cells, patch-clamp

## Abstract

Human pluripotent stem cell (hPSC)-derived retinal pigment epithelium (RPE) is extensively used in RPE research, disease modeling, and transplantation therapies. For successful outcomes, a thorough evaluation of their physiological authenticity is a necessity. Essential determinants of this are the different ion channels of the RPE, yet studies evaluating this machinery in hPSC-RPE are scarce. We examined the functionality and localization of potassium (K^+^) channels in the human embryonic stem cell (hESC)-derived RPE. We observed a heterogeneous pattern of voltage-gated K^+^ (K_V_) and inwardly rectifying K^+^ (Kir) channels. Delayed rectifier currents were recorded from most of the cells, and immunostainings showed the presence of K_V_1.3 channel. Sustained M-currents were also present in the hESC-RPE, and based on immunostaining, these currents were carried by KCNQ1-KCNQ5 channel types. Some cells expressed transient A-type currents characteristic of native human fetal RPE (hfRPE) and cultured primary RPE and carried by K_V_1.4 and K_V_4.2 channels. Of the highly important Kir channels, we found that Kir7.1 is present both at the apical and basolateral membranes of the hESC- and fresh native mouse RPE. Kir currents, however, were recorded only from 14% of the hESC-RPE cells with relatively low amplitudes. Compared to previous studies, our data suggest that in the hESC-RPE, the characteristics of the delayed rectifier and M-currents resemble native adult RPE, while A-type and Kir currents resemble native hfRPE or cultured primary RPE. Overall, the channelome of the RPE is a sensitive indicator of maturity and functionality affecting its therapeutic utility.

## Graphical Abstract

**Graphical Abstract:**
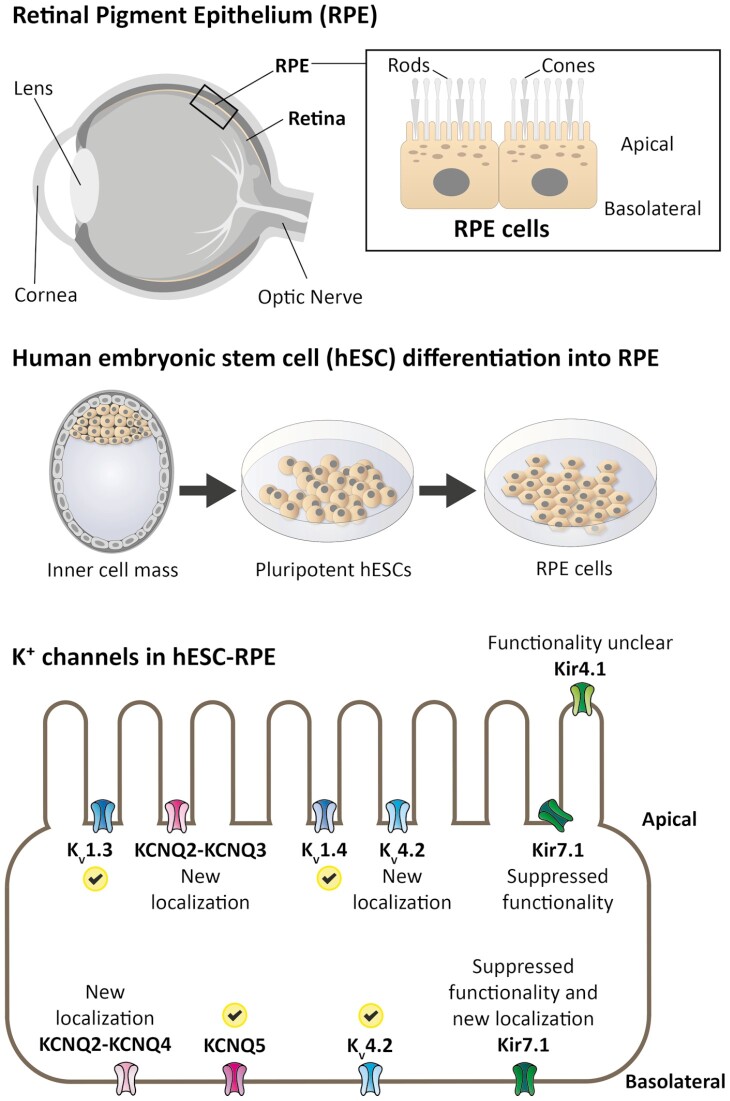

**Significance Statement**

Human pluripotent stem cell (hPSC)-derived retinal pigment epithelium (RPE) is an important cell source for research and therapy development demanding physiological validation for success. Ion channels regulate RPE physiology, and their alterations can lead to retinal diseases. Still, characterization of ion channels in the hPSC-RPE is in its infancy. This study focused on ubiquitous potassium (K^+^) channels involved in diverse physiological functions. Several types of functional K^+^ channels were discovered with similarities but also clear differences compared to the native RPE. This indicates promising functionality, yet raises issues essential for disease modeling and clinical applications.

## Introduction

Retinal pigment epithelium (RPE) in the back of the eye is critical for retaining our visual ability. Locating between the photoreceptors and the choroid,^1^ RPE transports ions, water, nutrients, and metabolites, phagocytoses photoreceptor outer segments, secrets growth factors, and supports the visual cycle.^2^ Ion channels have essential roles in these critical RPE functions,^2^ and potassium (K^+^) channels form one of the most prominent ion channel families in the RPE. Here we focus on the voltage-gated delayed rectifier K^+^ channels, KCNQ (K_V_7) channels, and A-type channels, as well as inwardly rectifying K^+^ (Kir) channels that have been widely studied in the cultured primary RPE and freshly isolated native RPE.

K^+^ channels in the RPE are linked to the generation of membrane potential,^3^ control of cell volume,^4^ transport of ions and water,^2^ and buffering of K^+^ concentration following its light-induced changes in the subretinal space.^5^ Recently, Kir7.1 was shown to participate in the phagocytosis of photoreceptor outer segments^6-8^ and secretion of growth factors.^7^ Due to the great importance of K^+^ channels to RPE functionality, understanding their physiology in human pluripotent stem cell (hPSC)-derived RPE is important for the success of cell transplantation therapies. These treatments have proceeded to clinical trials,^9-17^ yet, to our best knowledge, detailed K^+^ channel characterization of hPSC-RPE is still lacking. Furthermore, hPSC-RPE is being used in healthy and disease-specific cell models to study RPE cell biology and visual disorders.^6,8,18-30^ RPE participates in the pathogenesis of numerous diseases that threaten visual function, such as age-related macular degeneration (AMD),^31^ Bestrophinopathy,^32^ and Leber congenital amaurosis.^6,8^ Some of these diseases are directly linked to the misfunctioning of a specific ion channel. As an example of this, Kir7.1 readthrough therapy and gene augmentation has been investigated in Leber congenital amaurosis,^8^ where the loss of Kir7.1 function impairs the cell alignment^6^ and phagocytosis.^6,8^

In this study, we investigated the functionality and localization of the K^+^ channels in the human embryonic stem cell (hESC)-derived RPE. Our patch-clamp measurements and immunostainings reveal the presence of delayed rectifier K^+^ channels, KCNQ channels, A-type channels as well as Kir channels in the hESC-RPE. When compared to the literature, the features of the delayed rectifier currents and M-currents corresponded to the fresh native adult RPE, however, the amplitude and incidence of A-type and Kir currents resembled the native human fetal RPE (hfRPE) or cultured primary RPE.

## Materials and Methods

### hESC-RPE Culture and Sample Preparation

hESC lines Regea08/017, Regea08/023, and Regea11/013 were cultured and spontaneously differentiated in floating cell clusters as previously described.^33,34^ Shortly, the pigmented areas were manually separated and then dissociated using TrypLE Select (Invitrogen, UK). The isolated cells were seeded (5.5 × 10^5^ cells/cm^2^) onto collagen IV (5 μg/cm^2^, Sigma-Aldrich, St. Louis, MO) coated 24-well cell culture plates (Corning CellBIND; Corning, Inc., Corning, NY). This was followed by cryopreservation for the cell line Regea11/013.^35^ The pigmented cells were passaged for maturation on hanging culture inserts (2.5 × 10^5^ cells/cm^2^, polyethylene terephthalate (PET), pore size 1 μm, Merck Millipore or 7.5 × 10^4^ cells/cm^2^, PET transparent, pore size 1 µm, Sarstedt) treated with collagen IV (10 μg/cm^2^, Sigma-Aldrich) or with collagen IV and laminin (1.8 μg/cm^2^, LN521, Biolamina, Sweden). The cells were cultured for approximately 85-97 days until mature monolayers were obtained based on the cobblestone morphology, strong pigmentation, transepithelial resistance value over 200 Ω·cm^2^, and polarization of the RPE specific markers. For patch-clamp experiments, cells in the mature monolayers were dissociated with TrypLE Select (10 minutes incubation at 37 °C) and adhered to coverslips treated with poly-l-lysine (Sigma-Aldrich) for immediate measurements. For immunostaining, the insert membrane with the hESC-RPE monolayer was removed from the insert and cut into pieces.

### hfRPE Culture

The hfRPE cells were a generous gift from National Eye Institute, NIH (NEI), and the research of hfRPE cells followed the tenets of the Declaration of Helsinki and the NIH institutional review board. The cells were cultured as described before.^36^ The cells were seeded on 3 µg/cm^2^ human extracellular matrix (ECM)-coated (Corning, UK) inserts (pore size 1 µm, Merck Millipore, Germany) at a density of 10^6^ cells/cm^2^. The cells were cultured on the inserts for 48-58 days to confluent and mature monolayers. For the patch-clamp experiments, the cells were detached from the inserts with Trypsin (7 minutes incubation at 37 °C) (Lonza, Walkersville, MD) and measured similarly to hESC-RPE.

### Isolation of Mouse RPE

C57BL/6 mice at the age of 8-12 weeks were used in this study for immunostaining. The mice were euthanized by CO_2_ inhalation and cervical dislocation, after which the eyes were enucleated and bisected along the equator. The eyecups were sectioned and the retina removed in Ames’ solution (Sigma-Aldrich) with 10 mM HEPES, pH adjusted to 7.4 with NaOH.

### Ethical View

The National Authority for Medicolegal Affairs, Finland approved the research with human embryos (Dnro 1426/32/300/05). The Local Ethics Committee of the Pirkanmaa Hospital District, Finland, granted a supportive statement to derive and expand hESC lines from surplus embryos for research purposes (R05116). No new cell lines were derived in this study. Eye tissues from C57BL/6 mice were obtained after euthanization according to the protocols approved and monitored by the Animal Experiment Board of Finland. All handling and maintenance of the animals followed the ARVO Statement for the Use of Animals in Ophthalmic and Vision Research and the Finnish Animal Welfare Act 1986.

### Patch-Clamp Measurements and Analysis

Patch-clamp measurements were conducted at room temperature (RT) on single hESC-RPE and hfRPE cells in whole-cell configuration. Patch pipettes (resistance 4-8 MΩ) were filled with an internal solution containing (in mM): 25 KCl, 83 K-gluconate, 5.5 EGTA, 0.5 CaCl_2_, 4 ATP-Mg, 0.1 GTP-Na, 10 HEPES, 5 NaCl, and 2 lidocaine *N*-ethyl chloride (L1663; Sigma-Aldrich) to block the sodium currents.^37^ pH was adjusted to ~7.2 with KOH, and osmolarity was adjusted to ~290 mOsm with sucrose. In control conditions, the tissue was perfused with Ames’ solution (A1420, Sigma-Aldrich) supplemented with 10 mM HEPES and 10 mM NaCl or with Na^+^-based solution containing (in mM): 120 NaCl, 1.1 CaCl_2_, 1.2 MgCl_2_, 10 HEPES, 6 glucose, and 3 KCl. In both solutions, pH was adjusted to 7.4 with NaOH, and the osmolarity was set to ~305 mOsm with sucrose. For the enhancement of the Kir currents, Na^+^-based solution was modified by compensating NaCl with an equivalent amount of RbCl. In the pharmacological experiments, the bath solution contained general K^+^ channel blockers 5 mM Ba^2+^ or 20 mM tetraethylammonium chloride (TEA), or the following specific K^+^ channel modulators delayed rectifier K_V_1.3 inhibitor 10 nM Agitoxin-2 (RTA-420, Alomone) or KCNQ channel blocker 300 nM linopirdine (L134, Sigma-Aldrich).

The recordings were made in voltage-clamp mode using the Axopatch200B patch-clamp amplifier connected to an acquisition computer via AD/DA Digidata1440 (Molecular Devices, CA). The liquid junction potential (LJP) was negligible in Ames’ solution. In Na^+^-based and Rb^+^-based extracellular solutions LJP was measured to be 11 mV and 4 mV, respectively, and these values were taken into account in the data analysis. Access resistance was below 30 MΩ, and membrane resistance was above 200 MΩ. The membrane capacitance was 27 ± 1.2 pF (mean ± SEM, *n* = 73) for the hESC-RPE cells and 22 ± 1.5 pF (mean ± SEM, *n* = 9) for the hfRPE cells.

The M-current conductance was calculated using a tail current analysis as previously described.^3,38^ Shortly, starting from −10 mV holding potential, the membrane potential was stepped from −140 mV to 40 mV in 10 mV steps for 1000 ms. Tail currents appeared on return to −10 mV, and this conductance was determined as


$$\begin{array}{l} g_{{}_{K,-10mV}}=\frac{I_{K,-10mV}}{V_{m}-E_{K}} \end{array}$$ (1)

where *I*_K,−10 mV_ is the amplitude of the tail current at −10 mV that is divided by the driving force of K^+^ at −10 mV formed by the difference between the membrane potential (*V*_m_) and the K^+^ reversal potential (*E*_K_). The K^+^ conductance at each prepulse potential equals to


$$\begin{array}{l} g_{K}\left(V\right)=g_{max}-g_{{}_{K,-10mV}} \end{array}$$ (2)

that is the maximum conductance (*g*_max_) minus the conductance activated by returning from the prepulse potential to −10 mV. The nonlinear least-squares fit of the data to a Boltzmann equation was performed as follows


$$\begin{array}{l} \frac{g}{g_{max}}=\frac{1}{1+e^{\frac{V1/{}_{2}-V_{m}}{S}}} \end{array}$$ (3)

where *V*_½_ is the voltage at which the conductance is half-maximal, and *S* is the slope factor giving the steepness of the voltage dependence.

The incidence of a specific current type was calculated so that the number of cells detected with the current was divided by the total number of cells studied for that specific current type. Current-voltage (IV)-curves were obtained from the peak value of the current at given voltages using Python 3.8 and the pyABF module.^39^ The averaging, normalization, and statistical analysis were performed with Python 3.8. pandas, NumPy, sklearn, and scipy modules. For plotting the data, Origin software (OriginLab) was used. The data is stated as mean ± SEM (*n*, *p*), where *n* refers to the number of samples and *p* refers to statistical significance calculated using the Mann-Whitney *U*-test.

### Immunostaining and Confocal Microscopy

The immunofluorescence staining of hESC-RPE monolayers and mouse RPE eyecups, as well as their paraffin-embedded vertical sections, were done similarly to our previous study.^40^ Vertical sections of 7 μm in thickness were cut with a Leica SM2000 R sliding microtome (Leica Biosystems). In this study, we used primary antibodies Kir4.1 (1:100; ab80959; Abcam, UK), Kir7.1 (1:100; ab170631; Abcam), K_V_1.4 (1:50; ab99332; Abcam), K_V_4.2 (1:50; ab46797; Abcam), KCNQ1 (1:100; APC-022; Alomone Labs, Jerusalem, Israel), KCNQ2 (1:100; APC-050; Alomone Labs), KCNQ3 (1:100; APC-051; Alomone Labs), KCNQ4 (1:100; APC-164; Alomone Labs), KCNQ5 (1:100; APC-155; Alomone Labs), cellular retinaldehyde-binding protein (CRALBP; ab15051; 1:500; Abcam), zonula occludens (ZO-1; 339100; 1:50; Life Technologies), claudin-3 (1:80; 34-1700; Invitrogen, USA), Na^+^/K^+^-ATPase (1:200; ab7671; Abcam) and Bestrophin-1 (1:500; 016-Best1-01; Lagen Laboratories, USA) (see Supplementary Table S1). The secondary antibodies and phalloidins used here are listed in Supplementary Table S1. The nuclei were stained with the 4ʹ,6-diamidino-2-phenylindole (DAPI) included in the ProLong Gold antifade mounting medium (P36935; Thermo Fisher Scientific). The labeling specificity of the K^+^ channel antibodies used in the study was investigated in mouse tissue sections (see Supplementary Fig. S5).

Zeiss LSM780 laser scanning confocal microscope (LSCM) on an inverted Zeiss Cell Observer microscope (Zeiss, Jena, Germany) and Plan-Apochromat ×63/1.4 oil immersion objective were used for confocal microscopy. Voxel size was set to *x* = *y* = 66 nm and *z* = 200 nm and image size to 1024 × 1024 pixels. Images requiring more detailed analysis (Fig. 5) were denoised by deconvolution using Huygens Essential (SVI) software with theoretical PSF, signal-to-noise ratio of 5-19, and a quality threshold of 0.01. The refractive index of the sample was provided by the manufacturer of the ProLong Gold antifade mounting medium (Thermo Fisher Scientific). Confocal microscopy of the mouse tissue samples for antibody testing (Supplementary Fig. S5) was conducted using Nikon A1R laser scanning confocal microscope mounted in inverted Nikon Ti-E (Nikon Instruments Europe BV, Amsterdam, Netherlands) using a Plan-Apochromat 60×/1.4 oil immersion objective. The laser light intensity was adjusted to minimize photobleaching, and the detector sensitivity was adjusted for each sample to optimize the image brightness and to avoid saturation. Images were processed with ImageJ,^41^ where only linear adjustments to brightness and contrast were conducted, avoiding saturation. The final images were assembled using Adobe Photoshop CS6 (Adobe Systems, San Jose, USA).

## Results

### Maturity of the hESC-RPE

**Figure 1.**
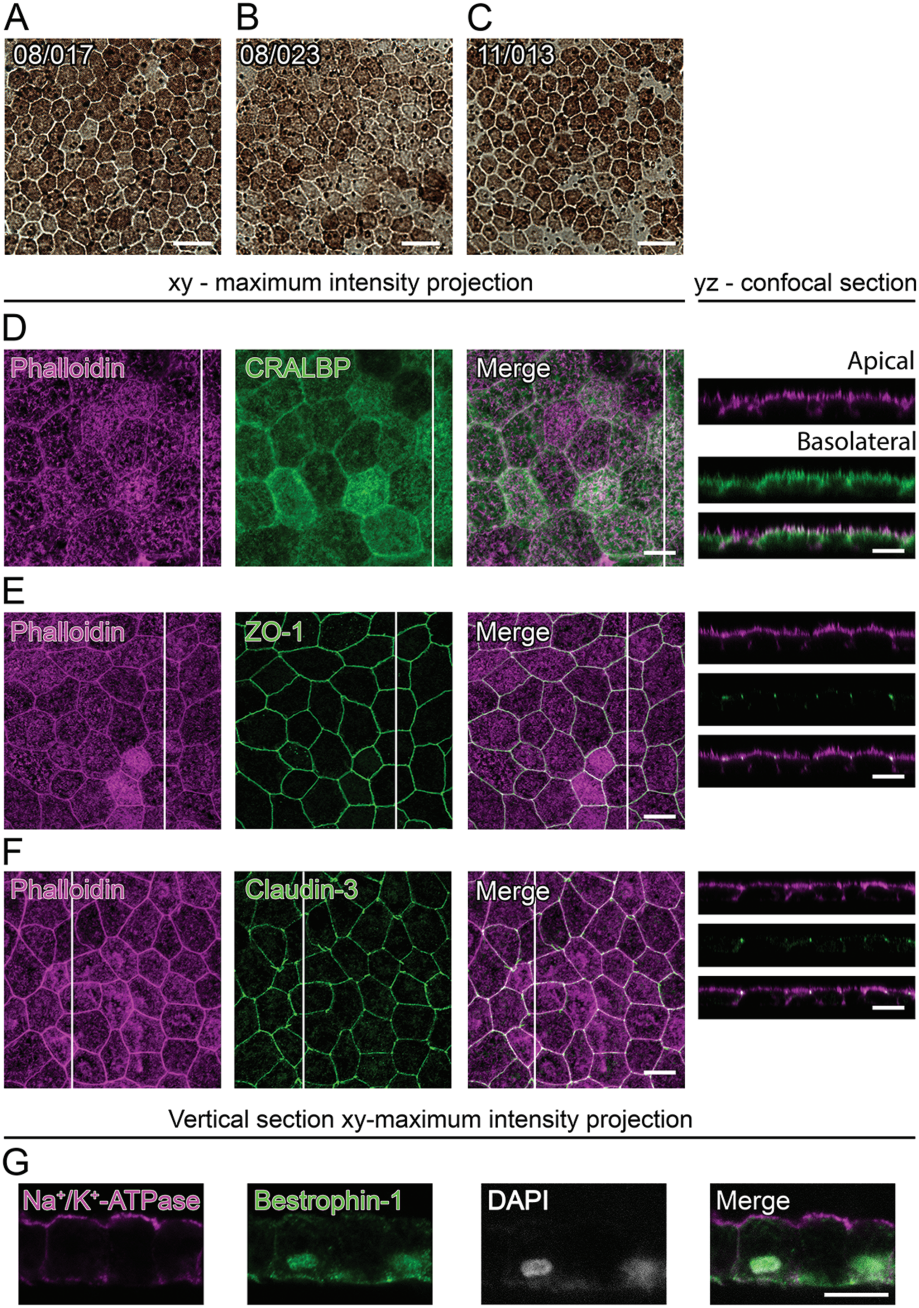
Morphology and key protein localization in the hESC-RPE. BF images of the monolayers from cell lines (**A**) Regea08/017, (**B**) Regea08/023, and (**C**) Regea11/013. Scale bars 20 µm. Xy-maximum intensity projections and yz-confocal sections (apical side upwards, localization of the section highlighted with a white bar) of the hESC-RPE monolayers. Actin cytoskeleton (phalloidin, magenta) labeled together with (**D**) cellular retinaldehyde-binding protein (CRALBP, green), and tight junction proteins (**E**) ZO-1 (green) and (**F**) claudin-3 (green). (**G**) *Xy*-maximum intensity projections (apical side upwards) of paraffin-embedded vertical sections with Na^+^/K^+^-ATPase (magenta) and Bestrophin-1 (green) labeled together with cell nuclei (DAPI, gray). Scale bars 10 µm. (D-G) show representative images of the cell lines 08/023 and 08/017. Abbreviations: BF, bright field; hESC, human embryonic stem cell; RPE, retinal pigment epithelium; CRALBP, cellular retinaldehyde-binding protein; ZO-1, Zonula occludens; DAPI, 4ʹ,6-diamidino-2-phenylindole.

Mature RPE monolayers derived from hESC lines Regea08/017 (Fig. 1A), Regea08/023 (Fig. 1B), and Regea11/013 (Fig. 1C) showed pigmentation and cobblestone morphology. In hESC-RPE, CRALBP (Fig. 1D), and Na^+^/K^+^-ATPase (Fig. 1G) were detected at the apical membrane, while Bestrophin-1 (Fig. 1G) was found primarily at the basolateral membrane of the monolayer. Typical for mature RPE,^42^ Zonula occludens (ZO-1) (Fig. 1E), and claudin-3 (Fig. 1F) localized to the cell-cell junctions together with phalloidin stained actin bands.

### Delayed Rectifier Currents

**Figure 2.**
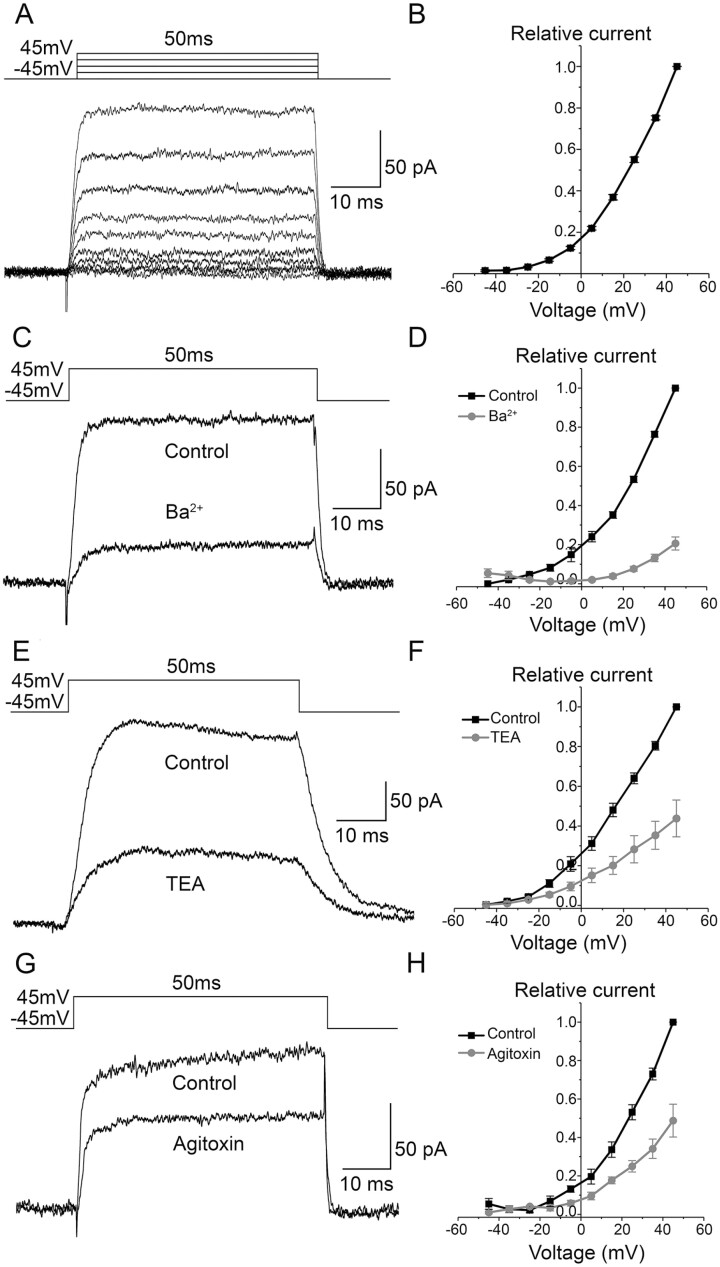
Delayed rectifier currents in the hESC-RPE. (**A**) An example of the delayed rectifier currents as responses to 50 ms voltage pulses from −45 mV to 45 mV in 10 mV increments and (**B**) the averaged and normalized IV-curve (mean ± SEM, *n* = 47). The effects of K^+^ channel inhibitors on the outward currents were studied at 45 mV with IV-curves showing the averaged and normalized values (mean ± SEM) using the following modulators: (**C**, **D**) 5 mM Ba^2+^ (*n* = 5), (**E**, **F**) 20 mM TEA (*n* = 7) and (**G**, **H**) 10 nM Agitoxin-2 (*n* = 4). Data has been pooled from the cell lines 08/023 and 08/017.

The resting membrane potential was recorded from 51 hESC-RPE cells with an average of −22 mV (values ranging from −10 mV to −36 mV). When bathed in control Ames’ solution, outward currents were recorded from 47 out of 59 (80%) hESC-RPE cells in response to a 50 ms voltage pulse from −45 mV to 45 mV in 10 mV increments (Fig. 2A). These currents activated at around −30 mV based on the normalized and averaged IV curve (Fig. 2B). The current amplitude at 45 mV was 92 ± 11 pA (*n* = 47). We tested the effects of general K^+^ channel blockers Ba^2+^ and TEA on the outward currents. At 45 mV potential, 5 mM Ba^2+^ decreased the currents by 79 ± 3% (*n* = 5, *P* < .05) (Fig. 2C, 2D) and 20 mM TEA by 56 ± 9% (*n* = 7, *P* < .05) (Fig. 2E, 2F). In addition, 10 nM Agitoxin-2, an inhibitor of the delayed rectifier channel K_V_1.3, decreased the currents by 51 ± 9% (*n* = 4, *P* < .05) (Fig. 2G, 2H). The presence of K_V_1.3 in hESC-RPE was confirmed by immunostaining (Supplementary Fig. S1A).

### Sustained M-currents

**Figure 3.**
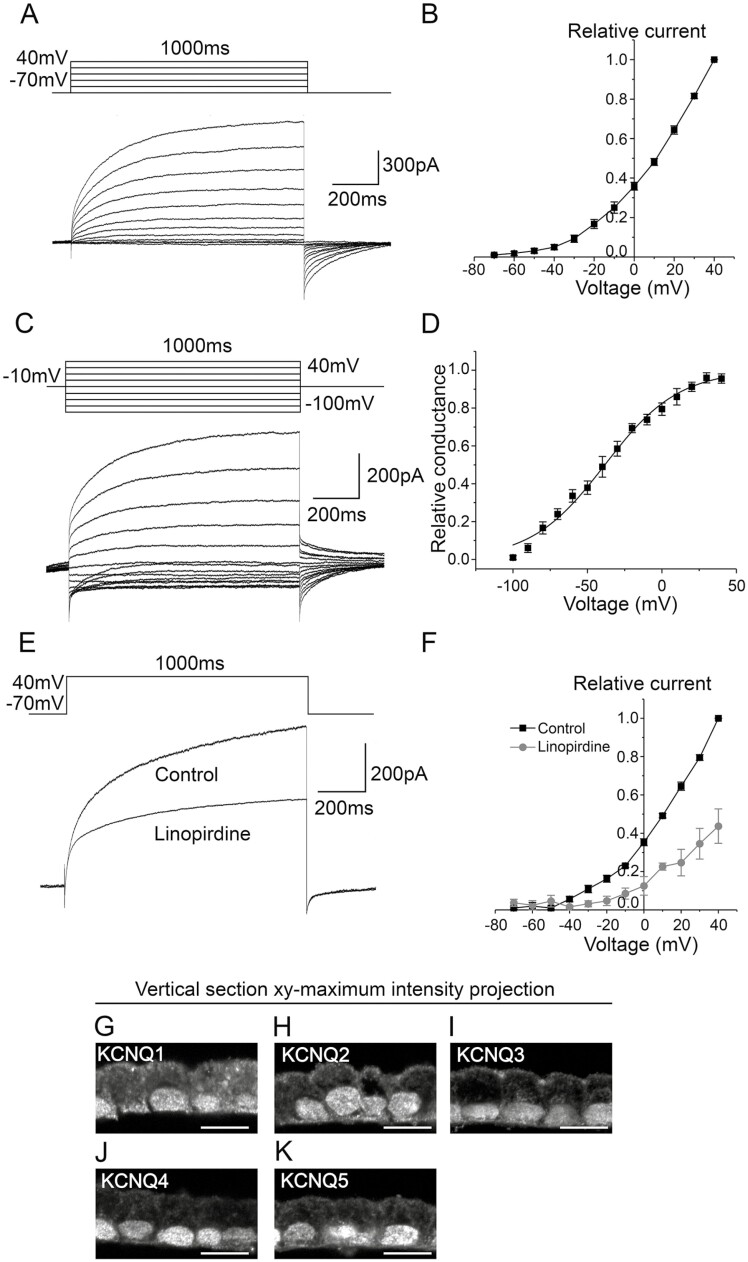
M-currents in the hESC-RPE. (**A**) Representative example of the sustained currents as responses to 1000 ms voltage steps from −70 mV to 40 mV in 10 mV increments (**B**) and the normalized and averaged IV-curve (mean ± SEM, *n* = 9). (**C**) Current responses evoked by 1000 ms voltage pulses from −10 mV holding potential to −100 mV and up to 40 mV test potentials in 10 mV steps (**D**) and the normalized and averaged GV-curve (mean ± SEM, *n* = 5) that has been obtained using tail current analysis (see Methods). (**E**) The effect of 300 nM linopirdine on the currents evoked by 1000 ms voltage pulses from −70 mV to 40 mV (**F**) and the averaged and normalized IV-curve (mean ± SEM, *n* = 3). Immunostainings of paraffin-embedded vertical sections shown as *xy*-maximum intensity projections (apical side upwards) for KCNQ channels (gray): (**G**) KCNQ1, (**H**) KCNQ2, (**I**) KCNQ3, (**J**) KCNQ4, and (**K**) KCNQ5. Scale bars 10 µm. (A-F) present patch-clamp data from the cell line 11/013 and (G-K) show representative images of the cell lines 08/023 and 08/017. Abbreviations: hESC, human embryonic stem cell; RPE, retinal pigment epithelium.

The outward currents were further studied with a prolonged pulse of 1000 ms from −70 mV to 40 mV in 10 mV steps. Of the total 41 measured hESC-RPE cells, 9 cells (22%) carried slowly activating and sustained currents (Fig. 3A). The currents activated at around −60 mV according to the normalized and averaged IV curve (Fig. 3B). The current amplitude at 40 mV was 529 ± 164 pA (*n* = 9). To determine the conductance, starting from −10 mV holding potential, the hESC-RPE cells were stepped from −100 mV to 40 mV in 10 mV steps (Fig. 3C). The normalized and averaged conductance-voltage (GV)-curve (Fig. 3D) was calculated by applying the tail current analysis (see Methods) using equations 1-3 resulting in the voltage with half-maximum conductance *V*_½_ = −37.2 ± 3.6 mV (*n* = 5) and slope factor *S* = 25.7 ± 2.5 (*n* = 5). The maximum conductance *g*_K, −10mV_ according to equation 1 was 1.8 nS ± 0.6 nS (*n* = 5). We used a KCNQ channel blocker linopirdine to characterize the slowly activating currents in the hESC-RPE. A 300 nM linopirdine decreased the current amplitude by 57 ± 10% (*n* = 3, *P* < .05) at the 40 mV voltage (Fig. 3E, 3F).

Immunostaining of paraffin-embedded vertical sections of the hESC-RPE was performed for KCNQ1-KCNQ5 channel subtypes. KCNQ1 was present in the hESC-RPE, however, its localization was inconclusive (Fig. 3G). KCNQ2 and KCNQ3 were detected at the apical and basolateral membranes of the hESC-RPE (Fig. 3H, 3I), and KCNQ3 stained the cell-cell junctions as well (Fig. 3I). KCNQ4 and KCNQ5 showed faint basolateral staining (Fig. 3J, 3K). Confocal images of KCNQ1-KCNQ5 in the hESC-RPE monolayer support this localization pattern (Supplementary Fig. S2A-S2E). Although several of the K^+^ channel antibodies also stain the nuclei and the presence of different ion channels in the nuclei has been demonstrated in the literature,^43^ we cannot rule out the nuclear staining shown in Fig. 3 and elsewhere in the study to be unspecific.

### Transient A-type Currents

**Figure 4.**
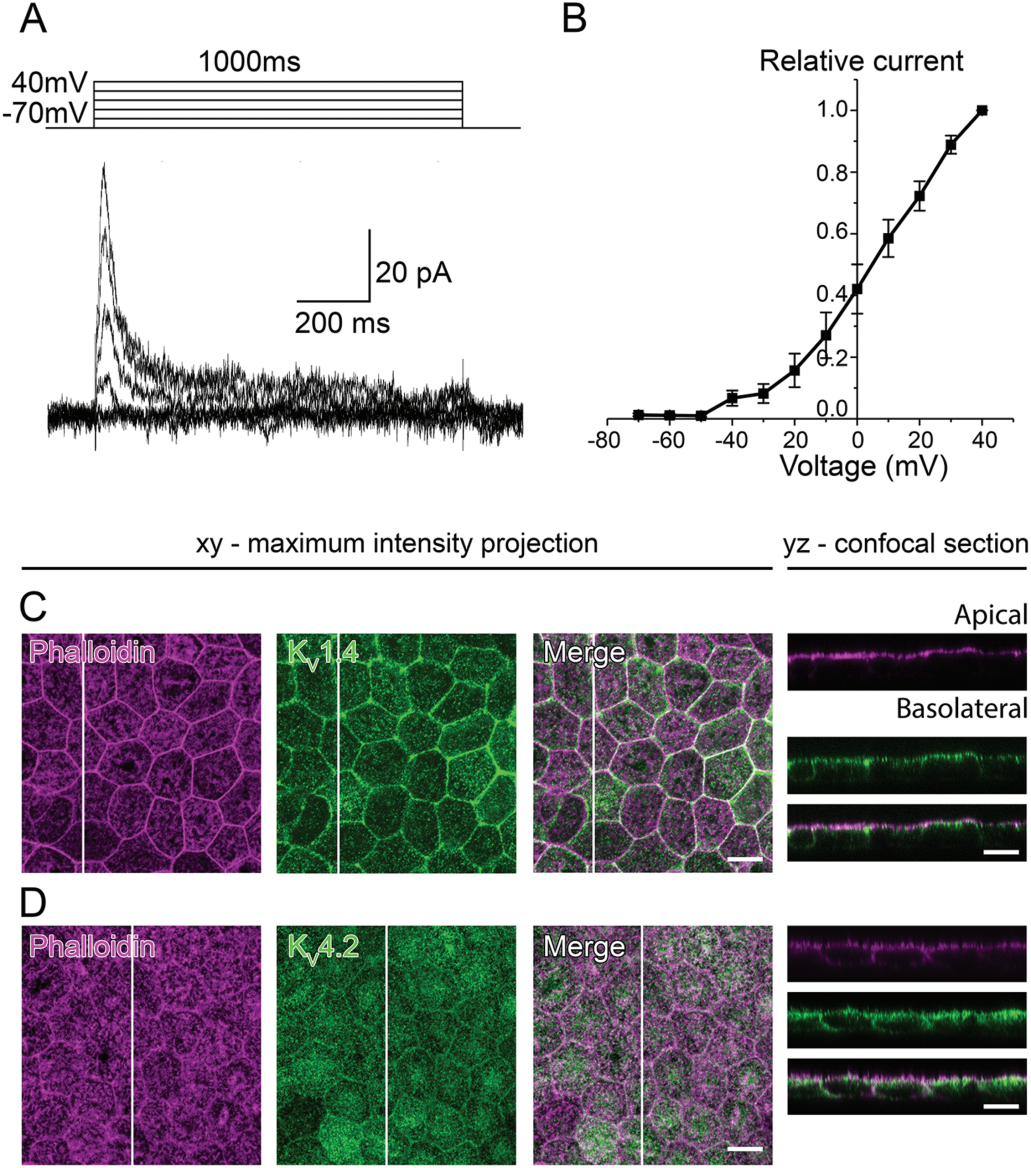
Transient A-type currents in the hESC-RPE. (**A**) Transient outward currents as responses to 1000 ms voltage pulses from −70 mV to 40 mV in 10 mV steps, and (**B**) the normalized and averaged IV-curve (mean ± SEM, *n* = 6). Confocal images of the hESC-RPE monolayers with *xy*-maximum intensity projections and *yz*-confocal sections (apical side upwards, localization of the section highlighted with a white bar). Actin cytoskeleton (phalloidin, magenta) stained with (**C**) K_V_1.4 (green) and (**D**) K_V_4.2 (green). Scale bars 10 µm. (A, B) present patch-clamp data from the cell line 11/013 and (C, D) show representative images of the cell lines 08/023 and 08/017. Abbreviations: hESC, human embryonic stem cell; RPE, retinal pigment epithelium; K_V_, voltage-gated K^+^ channel.

In addition to the slowly activating current, we found fast activating currents in 6 out of 41 tested hESC-RPE cells (15%) that inactivated completely during the prolonged pulse (Fig. 4A). The currents activated at −50 mV as seen in the normalized and averaged IV-curve (Fig. 4B), and their amplitude at 40 mV was 108 ± 26 pA (*n* = 6). The current pattern and the IV-curve closely resembled the A-type currents previously measured in the primary cultures of rabbit^44^ and human^45,46^ RPE or in the freshly isolated native hfRPE.^45^ Indeed, our immunostainings revealed the presence of K_V_1.4 and K_V_4.2 in the hESC-RPE. K_V_1.4 localized at the apical membrane and cell-cell junctions (Fig. 4C), while K_V_4.2 labeled both the apical and basolateral membranes without prominent junctional localization (Fig. 4D).

### Inwardly Rectifying K^+^ (Kir) Currents

**Figure 5.**
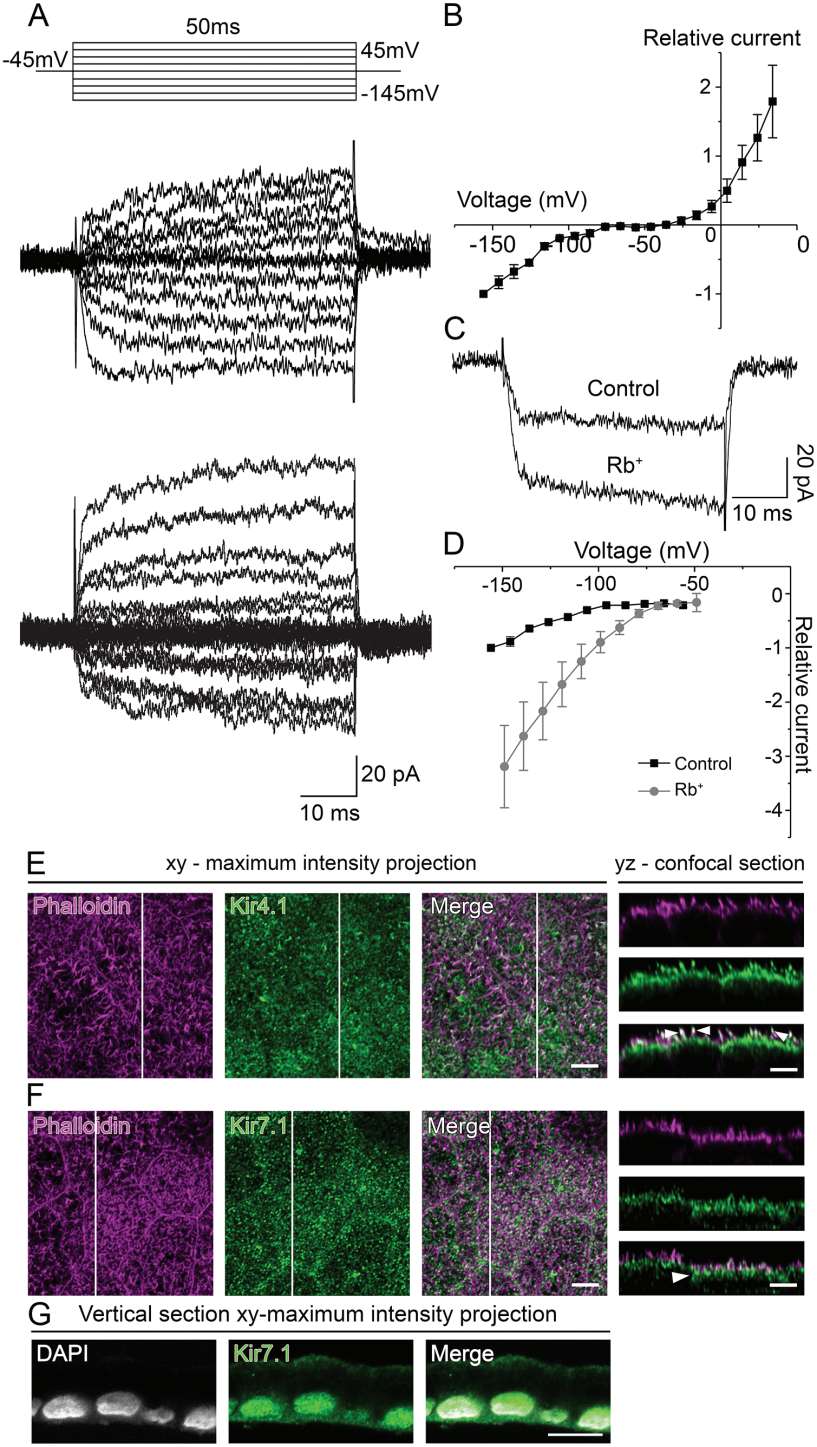
Inwardly rectifying K^+^ (Kir) channels in the hESC-RPE. (**A**) Two examples of Kir currents in the hESC-RPE as responses to 50 ms voltage pulses starting from −45 mV holding potential and stepping first to −145 mV and in 10 mV intervals to 45 mV. (**B**) Normalized and averaged IV-curve (mean ± SEM, *n* = 5). (**C**) The effect of replacing extracellular Na^+^ by Rb^+^ on Kir currents elicited by a 50 ms voltage step from −45 mV to −145 mV (**D**) with the IV-curve showing averaged and normalized data (mean ± SEM, *n* = 8). Localization of Kir channels in the hESC-RPE monolayers: actin cytoskeleton (phalloidin, magenta) labeled together with (**E**) Kir4.1 (green) and (**F**) Kir7.1 (green) and represented as *xy*-maximum intensity projections and *yz*-confocal sections (apical side upwards, localization of the section highlighted with a white bar). White arrows highlight the localization of Kir4.1 high in the microvilli and Kir7.1 at the root of the microvilli. Scale bars 5 µm. (**G**) Vertical section *xy*-maximum intensity projection (apical side upwards) of Kir7.1 (green) together with cell nuclei (DAPI, gray). Scale bar 10 µm. Data have been pooled from the cell lines 08/023 and 08/017. Abbreviations: hESC, human embryonic stem cell; RPE, retinal pigment epithelium; Kir, inwardly rectifying K^+^ channel; DAPI, 4ʹ,6-diamidino-2-phenylindole.

In control Na^+^-based solution, hyperpolarization of the hESC-RPE cells from −45 mV to −145 mV and depolarization from −45 mV to 45 mV in 10 mV steps revealed inwardly rectifying currents in 9 of the measured 64 cells (14%) (Fig. 5A). In the normalized and averaged IV-curve, the inwardly rectifying currents activated at near −70 mV (Fig. 5B). The maximum current amplitude at −145 mV was −43 ± 5 pA (*n* = 9). We replaced the Na^+^-based control solution with Rb^+^-based test solution to enhance the inwardly rectifying currents. Rb^+^ in the bathing solution successfully increased the amplitudes of the inwardly rectifying currents from the control value by 319 ± 76% (*n* = 8, *P* < .05) measured at −145 mV (Fig. 5C, 5D; Supplementary Fig. S4 shows full response families and IV curves in both conditions in one cell). Similar results were obtained in control Ames’ solution replaced with the Rb^+^-test solution (data not shown).

Immunostaining of the hESC-RPE monolayers showed that Kir4.1 (Fig. 5E) and Kir7.1 (Fig. 5F) both localize to the apical membrane so that Kir4.1 was detected especially at the apical microvilli and Kir7.1 at the root of the microvilli (white arrows in Fig. 5E and F). Immunostaining of paraffin-embedded vertical sections of Kir7.1 confirmed its apical localization and revealed its basolateral appearance as well (Fig. 5G).

### Localization of the K^+^ Channels in the Mouse RPE

**Figure 6.**
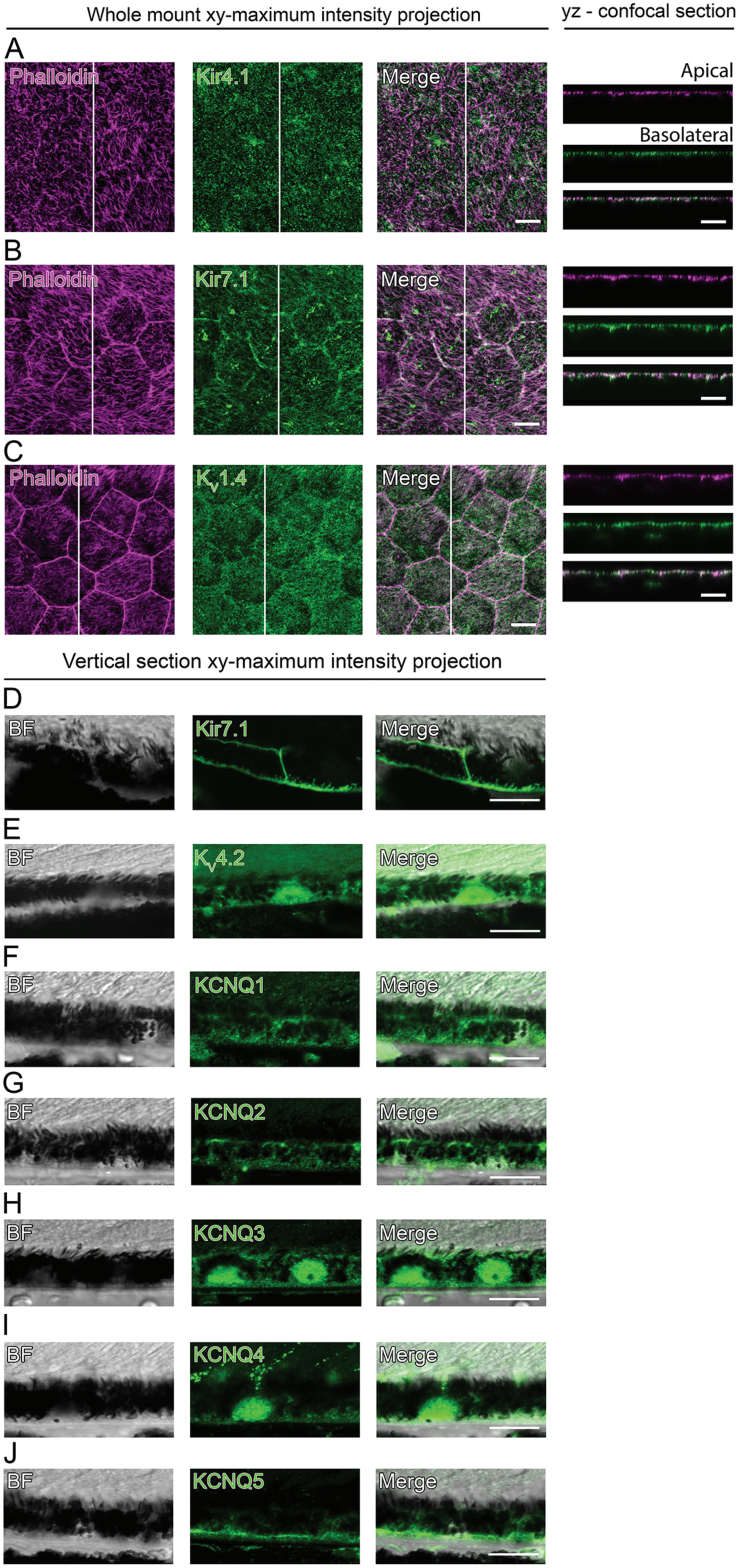
Localization of the K^+^ channels in the mouse RPE. Confocal images of the mouse RPE-eyecup whole mount preparations illustrate the *xy*-maximum intensity projections and *yz*-confocal sections (apical side upwards, localization of the section highlighted with a white bar), where actin cytoskeleton (phalloidin, magenta) is stained together with (**A**) Kir4.1 (green), (**B**) Kir7.1 (green), and (**C**) K_V_1.4 (green). Confocal images of paraffin-embedded vertical sections of mouse eyecups shown as *xy*-maximum intensity projections (apical side upwards), where BF images are presented together with the K^+^ channels (green). (**D**) Kir7.1, (**E**) K_V_4.2, (**F**) KCNQ1, (**G**) KCNQ2, (**H**) KCNQ3, (**I**) KCNQ4, and (**J**) KCNQ5. Scale bars 10 µm. Abbreviations: BF, bright field; Kir, inwardly rectifying K^+^ channel; K_V_, voltage-gated K^+^ channel.

To compare the localization of the K^+^ channels between hESC-RPE and native mouse RPE, we conducted immunostainings of the mouse RPE-eyecup whole-mount preparations and vertical sections of paraffin-embedded eyecups. Identical to the hESC-RPE, Kir4.1 (Fig. 6A) and Kir7.1 (Fig. 6B) were localized to the apical membrane in the mouse RPE. Interestingly, Kir7.1 showed a strong apical and basolateral staining in the mouse RPE (Fig. 6D), as had been observed in the hESC-RPE with weaker staining intensity (Fig. 5G). The localization of KCNQ1-KCNQ5 in the mouse RPE (Fig. 6F-6J, Supplementary Fig. S2F-S2J) followed the pattern seen in the hESC-RPE with the exception that KCNQ1 localized on the apical and basolateral membranes (Fig. 6F), KCNQ3 was not observed in cellular junctions (Fig. 6H, Supplementary Fig. S2H), and KCNQ5 formed especially strong basolateral staining (Fig. 6J). K_V_1.4 (Fig. 6C) localized to the apical cell membrane, and K_V_4.2 to the basolateral cell membrane (Fig. 6E) in the mouse RPE without the junctional localization of K_V_1.4 and apical localization of K_V_4.2 detected in the hESC-RPE.

### Outward Currents in the hfRPE

Using 50 ms voltage pulses from −45 mV to 45 mV in 10 mV steps, we observed outward currents in 11 of the 14 tested hfRPE cells (79%) (Supplementary Fig. S3A) with current amplitudes 168 ± 74 pA (*n* = 11). According to the normalized and averaged IV curve, the currents activated at around −30 mV (Supplementary Fig. S3B). Inwardly rectifying K^+^ currents were not detected in the 14 measured hfRPE cells.

## Discussion

hPSC-RPE shows great promise to be used in disease modeling^6,8,18-30^ and in transplantation therapies^9-17^ with their demonstrated expression of typical RPE markers^30,47,48^ and ability to perform key RPE functions such as phagocytosis,^6-8,29,30,37,40^ growth factor secretion^7,40,47^ and visual cycle.^49,50^ Cellular components essential for proper RPE physiology include ion channels as well. The knowledge about ion channels in hPSC-RPE has grown over the past few years,^6-8,18,22,23,28,37,40^ yet there is only limited information about the K^+^ channels in these cells.^6-8^ The need for a detailed characterization of the different ion channels in hPSC-RPE is highly essential. First, it is important to understand the functional capacity of the stem cell-derived RPE as a cell model to be used in investigations of both RPE physiology and RPE-related diseases. Second, for the success of transplantation therapies, it is critical to evaluate whether the cells resemble their native counterparts and are able to perform the critical RPE functions. To promote these goals, we showed the presence and proper functionality of several types of K^+^ channels in the hESC-RPE. With certain channel types, however, these cells demonstrated features that differed from the fresh native adult RPE.

In our study, the identification of the channel types was largely based on patch-clamp electrophysiology and analysis of 3 different hESC-RPE cell lines with no clear differences between the lines. We categorized the recorded currents based on their characteristics and drug sensitivity, and we further analyzed their incidence and amplitude as well. The main current type detected from most of the hESC-RPE cells was the outward K^+^ current. Comparable outward currents resembling delayed rectifiers have previously been measured from freshly isolated native RPE and cultured primary RPE from different species,^44,45,51-57^ including humans.^45^ In these publications, the currents appeared in the majority of the recorded cells,^44,45,54-57^ activated on average at −30 mV,^44,45,51-57^ and were sensitive to extracellular Ba^2+44,51,54^ and TEA.^44,45,54-56^ Agitoxin-2 was reported to inhibit the delayed rectifier current, and thus, it was identified as a subtype K_V_1.3^53,57^ that was also confirmed by immunostaining.^57,58^ In the hESC-RPE, the activation potential and the sensitivity to Ba^2+^, TEA, and Agitoxin-2 agreed with the previous studies.^44,45,51-57^ In addition, our immunostainings showed the presence of K_V_1.3 in the hESC-RPE comparable to the native mouse RPE. K_V_1.3 is regulated, for example, by tyrosine kinases and it may act as a functional antagonist of the voltage-gated L-type Ca^2+^ channels.^53^

Large and sustained M-currents have been detected in the cultured human primary RPE^46^ and in the freshly isolated native turtle,^54^ bovine,^56^ human,^3^ and monkey^38,59^ RPE. In the monkey RPE, KCNQ4 and KCNQ5 were considered as the main contributors to the current.^38^ The M-currents in the RPE have been shown to be sensitive to K^+^ channel blocker Ba^2+,3,56^ and KCNQ channel blockers linopirdine^38^ and XE991,^59^ but relatively insensitive to TEA.^3,38,54,56^ We recorded similar slowly activating currents with large amplitudes from the hESC-RPE with activation potential and conductance values comparable to the literature.^3,38^ The current amplitudes were decreased by linopirdine, as previously shown in RPE.^38^ However, the slope of the conductance curve was less steep than previously presented,^3,38^ and this may indicate that other ion channels contribute to the sustained current in the hESC-RPE, such as BK channels^60,61^ or voltage-gated chloride channels.^2,62^ The occurrence of the M-currents in the human RPE has varied from 6% in the cultured RPE^46^ and 26% in the native fetal RPE^45^ to almost 80% in the native adult RPE.^3^ Approximately 22% of the hESC-RPE cells conducted the M-currents that is close to the native hfRPE.

There is only limited information in the literature about the KCNQ transcripts, proteins, or membrane localization, and to our knowledge, this information is especially missing from the human RPE. RT-PCR analysis has revealed the expression of KCNQ1, KCNQ4, and KCNQ5 in the native bovine^63^ and monkey^59^ RPE, but only KCNQ5 was detected at the protein level in these cells.^59,63^ Based on the immunostainings, KCNQ5 was shown to localize to the basolateral membrane of the native monkey^59^ and rat^64^ RPE, and we confirmed this in our immunostainings with the hESC- and native mouse RPE. Furthermore, auxiliary subunit KCNE1 has been shown to localize to the apical and basolateral membranes of the native bovine RPE, where it may modify the surface expression and functional properties of the alpha subunits.^63^ In addition to KCNQ5, our immunostainings showed the presence of KCNQ1-KCNQ4 in both hESC-RPE and native mouse RPE. This supports the earlier RT-PCR data on the expression of KCNQ1 and KCNQ4 in the RPE^59,63^ and further suggests the presence of KCNQ2-3 as well. KCNQ channels are regulated by calmodulin,^64^ cell volume,^4^ and G-protein coupled receptors,^2^ and they participate in the generation of the membrane potential^3^ and control of the cell volume^4^ that are closely connected to the transport of ions and water.^2^ Therefore, KCNQ channels are an important target to investigate in relation to retinal degenerative diseases.^64^

Our patch-clamp recordings also revealed fast activating transient currents that inactivated during the prolonged stimulation. They resembled the A-type currents previously measured in the rabbit^44^ and human^45,46^ primary RPE cultures or the freshly isolated native hfRPE^45^ by their voltage to current relationship as well as by their activation and inactivation kinetics. These features may vary between different species and tissues.^65^ The subtype K_V_1.4 has been detected in the apical microvilli and K_V_4.2 in the basolateral membrane of the native mouse RPE^58^ and we confirmed this result in our immunostainings with mouse. However, in the hESC-RPE, we found K_V_1.4 at the apical membrane and cellular junctions, and K_V_4.2 both at the apical and basolateral membranes. Despite their membrane expression, functional A-type currents have not been measured in the fresh native adult RPE before. Interestingly, Wen et al found transient A-type currents almost in every measured native hfRPE cell and in 33% of the cultured human primary RPE cells, while these currents were not found from the native adult human RPE.^45^ They concluded that the membrane channel phenotype changes during maturation since the A-type currents disappear when the RPE cells mature. We detected A-type currents in 15% of the measured hESC-RPE cells that otherwise showed markers typical to mature RPE. This may indicate that the hESC-RPE expresses a phenotype that resembles the hfRPE, as previously suggested in the literature.^66-68^

Mild inwardly rectifying Kir7.1 currents have been recorded in the native and cultured RPE of different species,^5,8,44-46,51,52,54,55,57,69-72^ including humans.^8,45,46^ The currents were detected in almost all freshly isolated native human^45,46^ and monkey^45^ RPE cells, but they were mostly absent from the native hfRPE cells,^45^ and appeared to some extend (5-41%) in the primary RPE cultures.^44,45,52,57^ The mild inwardly rectifying current was reported to activate approximately at −70 mV with current amplitudes of hundreds of picoamperes.^44-46,51,52,54,55,57,69,70,72^ Interestingly, detectable but somewhat small (on average 144 pA) Kir7.1 currents were recently measured from the human-induced pluripotent stem cell (hiPSC)-derived RPE.^8^ Similarly, we detected small currents with inward rectification activating at around −70 mV in the hESC-RPE, yet in only 15% of the measured cells. The current amplitudes increased by Rb^+^ application, which is characteristic of the Kir7.1 channels.^8,70,73^ It is notable that the observed low amplitudes in both hiPSC-^8^ and hESC-RPE cannot be explained, for example, by the differences in the cell volume between the species when compared to previous studies from the native or cultured RPE. Therefore, we suggest that the observations indicate an attenuated Kir machinery in the hPSC-RPE compared to the native adult RPE.

Kir7.1 transcript and protein product have been detected in the bovine^74^ and human RPE^75^ with localization to the apical cell membrane in several cell types, including hPSC-RPE.^5-8,63,74,76^ Our immunostainings showed the apical localization of Kir7.1 in the hESC- and native mouse RPE, but, contrary to the literature, we also found Kir7.1 at the basolateral membrane of both RPE types. It is possible that the strong pigmentation has hindered the detection of the basolateral localization before, and its physiological relevance requires further investigation. Previously, Kir7.1 has been shown to colocalize with Na^+^/K^+^-ATPase, thus supporting its function in recycling K^+^ at the apical membrane.^74,76^ The apical localization of Kir7.1 enables the RPE to respond to the light-induced decrease in K^+^ concentration from 5 mM to 2 mM near the photoreceptors in the subretinal space.^5^ Typical to Kir7.1, its conductance increases with decreasing extracellular K^+^ concentration.^46,69,70^ It is also worth noting that Kir7.1 is regulated by intracellular ATP^71,72^ and pH.^73,77^ Interestingly, recent studies using the hiPSC-RPE cell model suggested the participation of Kir7.1 in the phagocytosis of photoreceptor outer segments^6-8^ and in the secretion of growth factors.^7^

Immunostainings have revealed the presence of Kir4.1 on the apical processes of the native rat^76,78^ and hiPSC-RPE,^7^ while Kir4.1 transcript has been absent in native bovine^74^ and human^79^ RPE. Kir4.1 currents have been measured only in the native rat RPE^78^ and cultured human primary RPE,^46^ while on the contrary, Kir7.1 currents have been demonstrated in several studies with cultured and native RPE.^5,8,44-46,51,52,54,55,57,69-72^ It has been speculated that even though Kir4.1 channels seem to be present in the RPE, their contribution to the macroscopic whole cell current is minor compared to Kir7.1.^70^ Our results support this as we found Kir4.1 on the apical processes of the hESC-RPE, yet we were not able to verify currents characteristic to Kir4.1. This can be influenced by culturing conditions as it has been shown in astrocytes that high glucose concentration during cell culture can remarkably reduce the functional expression of Kir4.1.^80^ In glucose concentrations typical to DMEM used in this study, a 50% reduction of Kir4.1 mRNA and protein expression was observed in astrocytes compared to cells grown in 5mM glucose.^80^

Our work shows that the hESC-RPE cells express a heterogeneous pattern of K^+^ channels with substantial variation in the channel profiles between the cells. Whether this indicates that RPE cells have functional heterogeneity allocating its functions between individual cells, is an intriguing possibility and question for further studies. Ion channels are sensitive indicators of RPE maturity^40,48,81^ and functionality,^6,37,40^ and the cell culturing conditions may also influence the channelome of the RPE. In native rabbit Müller cells, Kir4.1 expression first disappears when the cells are cultured but reappears following culturing on laminin-coated dishes in the presence of insulin.^82^ Interestingly in epithelia, the extracellular calcium level has been shown to affect the localization of KCNQ1.^83^ It is also worth bearing in mind that in culture, epithelial cells do not encounter physiological trans-epithelial ion gradients or their naturally occurring changes. Furthermore, the stem cell-derived tissue requires typically a long cultivation time to differentiate and mature. Therefore, it is topical to evaluate the present cell culturing methods and develop them further to meet the physiologically relevant requirements. It is possible that the current culture conditions affect the partially compromised functionality of the K^+^ channels observed in this study in the hESC-RPE.

## Conclusion

Our study revealed the presence of a diverse set of functional K^+^ channels in the hESC-RPE that is promising for the successful use of hPSC-RPE as cell models and in transplantation therapies. Our work highlights the importance of physiological evaluation to complement the gene and protein expression analyses. In the case of hESC-RPE, the K^+^ channel machinery related to A-type and Kir currents did not fully meet the features of the fresh native adult RPE. This influences the physiology of the hPSC-RPE and may reflect the sensitivity of ion channels to cell maturity and culture conditions requiring further investigations.

## Supplementary Material

szac029_suppl_Supplementary_Material

## Data Availability

The data that support the findings of this study are available from the corresponding author upon reasonable request.
