# Supplementary material for: Heterogeneity of Potassium Channels in Human Embryonic Stem Cell-Derived Retinal Pigment Epithelium
Source: Stem Cells Transl Med. 2022 May 27;11(7):753–66. doi: 10.1093/stcltm/szac029 (PMC9299513; doi:10.1093/stcltm/szac029)
Supplement: szac029_suppl_Supplementary_Material [file szac029_suppl_supplementary_material.pdf]

Supplementary material

Heterogeneity of Potassium Channels in Human Embryonic Stem Cell-Derived Retinal Pigment Epithelium

Iina Korkka[a], Heli Skottman[a], and Soile Nymark[a*]

[a]BioMediTech, Faculty of Medicine and Health Technology, Tampere University, Arvo Ylpön katu 34, 33520 Tampere, Finland

[*]Corresponding author: Soile Nymark, PhD, Arvo Ylpön katu 34, 33520 Tampere, Finland

Telephone: +358 40 849 0009, E-mail: soile.nymark@tuni.fi

Contents:

Supplementary Table S1. Details of the antibodies

Supplementary Figure S1. Immunostaining of $K_V1.3$ in the hESC- and mouse RPE

Supplementary Figure S2. Localization of KCNQ1-KCNQ5 in the hESC- and mouse RPE

Supplementary Figure S3. Outward currents in the hfRPE

Supplementary Figure S4. The effect of replacing extracellular Na^+ by Rb^+ on Kir currents

Supplementary Figure S5. Specificity of K^+ channel antibodies

Supplementary Table S1. Details of the antibodies. The antibody name, company, catalog number, and dilution ratio are listed for each primary and secondary antibody as well as for phalloidins used in this study. Abbreviations: CRALBP, cellular retinaldehyde-binding protein; Kir, inwardly rectifying K^+ channel; K_V, voltage-gated K^+ channel; ZO-1, Zonula occludens.

**Antibody**	**Company**	**Catalog number**	**Dilution**
Bestrophin-1	Lagen laboratories	016-Best1-01	1:500
claudin-3	Invitrogen	34-1700	1:80
CRALBP	Abcam	ab15051	1:500
Na^+/K^+-ATPase	Abcam	ab7671	1:200
Kir4.1	Abcam	ab80959	1:100
Kir7.1	Abcam	ab170631	1:100
K_V1.3	Alomone Labs	APC-002	1:100
K_V1.4	Abcam	ab99332	1:50
K_V4.2	Abcam	ab46797	1:50
KCNQ1	Alomone Labs	APC-022	1:100
KCNQ2	Alomone Labs	APC-050	1:100
KCNQ3	Alomone Labs	APC-051	1:100
KCNQ4	Alomone Labs	APC-164	1:100
KCNQ5	Alomone Labs	APC-155	1:100
ZO-1	Life Technologies	339100	1:50
Donkey anti-rabbit Alexa Fluor 488	Thermo Fisher Scientific	A-21206	1:200
Donkey anti-mouse Alexa Fluor 488	Thermo Fisher Scientific	A-21202	1:200
Donkey anti-mouse Alexa Fluor 568	Thermo Fisher Scientific	A10037	1:200
Donkey anti-mouse Alexa Fluor 647	Thermo Fisher Scientific	A31571	1:200
Donkey anti-goat Alexa Fluor 568	Thermo Fisher Scientific	A11057	1:200
Goat anti-rabbit Alexa Fluor 488	Thermo Fisher Scientific	A11008	1:200

Goat anti-rabbit Alexa Fluor 568	Thermo Fisher Scientific	A11011	1:200
Goat anti-mouse Alexa Fluor 488	Thermo Fisher Scientific	A11029	1:200
Goat anti-mouse Alexa Fluor 568	Thermo Fisher Scientific	A11031	1:200
Goat anti-mouse Alexa Fluor 647	Thermo Fisher Scientific	A21236	1:200
Phalloidin-Atto 633	Sigma-Aldrich	68825	1:100
Phalloidin tetramethylrhodamine B 568	Sigma-Aldrich	P1951	1:400
Alexa Fluor 647 phalloidin	Thermo Fisher Scientific	A22287	1:50

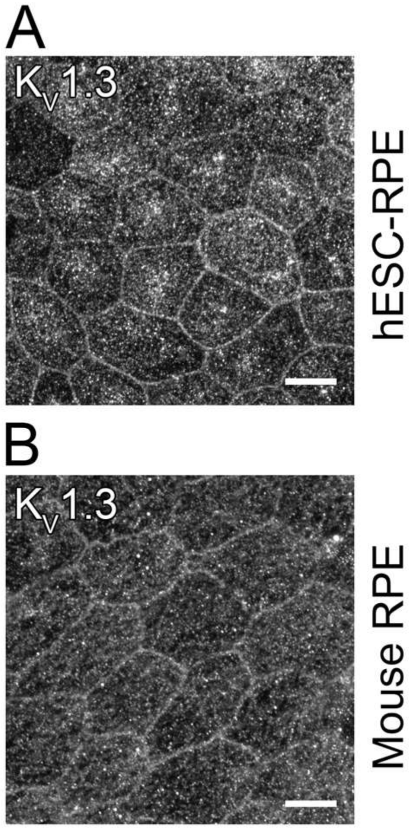

Supplementary Figure S1. Immunostaining of $K_V1.3$ in the hESC- and mouse RPE. Xy-maximum intensity projections of confocal images with $K_V1.3$ (gray) labelled in (A) hESC-RPE monolayer (representative of cell lines 08/023 and 08/017) and (B) mouse RPE-eyecup whole mount preparation. Scale bars 10 µm.

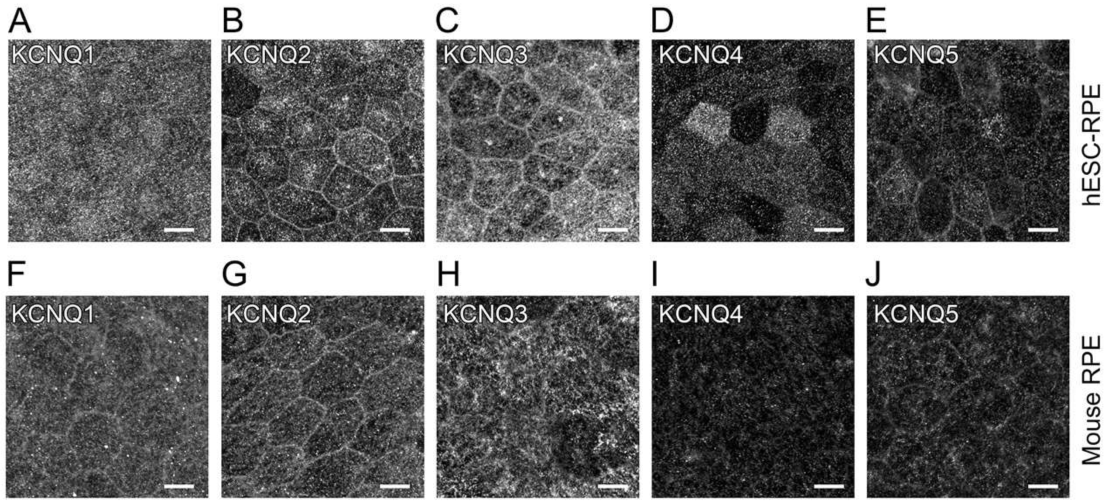

Supplementary Figure S2. Localization of KCNQ1-KCNQ5 in the hESC- and mouse RPE. Immunostainings represented as xy-maximum intensity projections of confocal images with KCNQ channels (gray) labeled in the hESC-RPE monolayers: (A) KCNQ1, (B) KCNQ2, (C) KCNQ3, (D) KCNQ4, and (E) KCNQ5. Similar labeling in the mouse RPE-eyecup whole mount preparations: (F) KCNQ1, (G) KCNQ2, (H) KCNQ3, (I) KCNQ4, and (J) KCNQ5. Scale bars 10 µm. (A-E) show representative images of the cell lines 08/023 and 08/017.

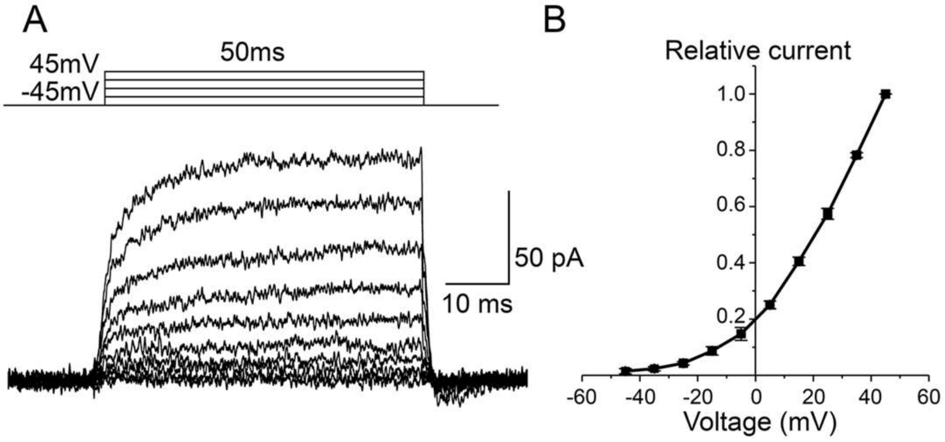

Supplementary Figure S3. Outward currents in the hfRPE. (A) An example of outward currents in the hfRPE elicited by 50 ms voltage pulses from −45 mV to 45 mV in 10 mV increments (B) and the normalized and averaged IV-curve (mean ± SEM, n = 11). Abbreviations: hfRPE, human fetal RPE.

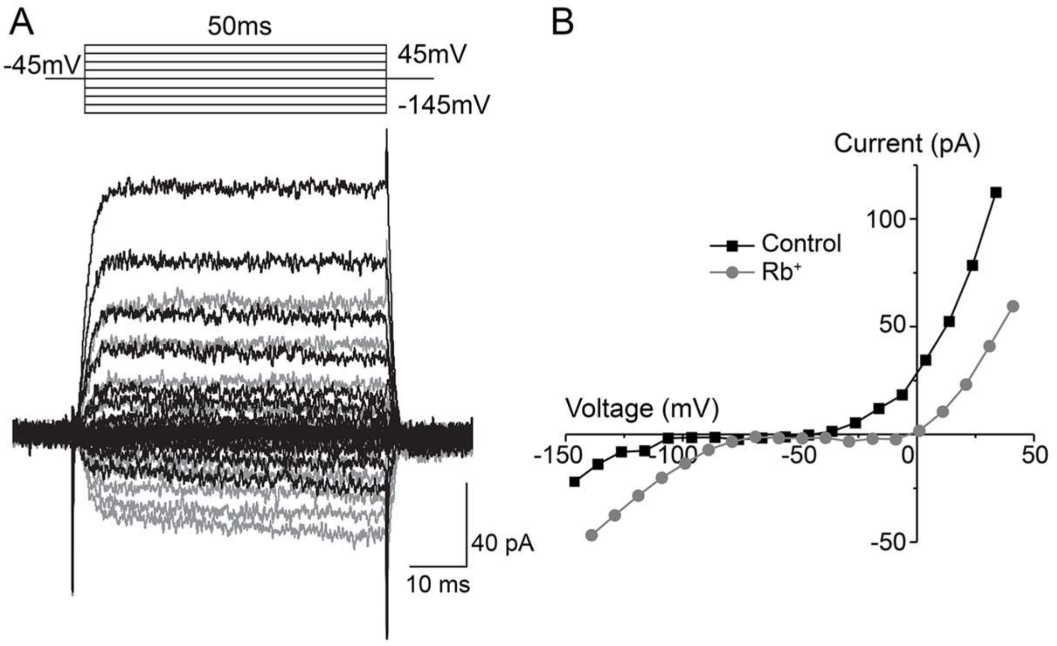

Supplementary Figure S4. The effect of replacing extracellular Na^+ by Rb^+ on Kir currents. (A) Examples of currents in hESC-RPE to a series of 50 ms voltage pulses starting from -45 mV holding potential and stepping first to -145 mV and then in 10 mV intervals to 45 mV. Currents in control solution are shown in black and currents after replacing extracellular Na^+ by Rb^+ (120 mM) in gray. (B) IV curves of the recordings shown in (A).

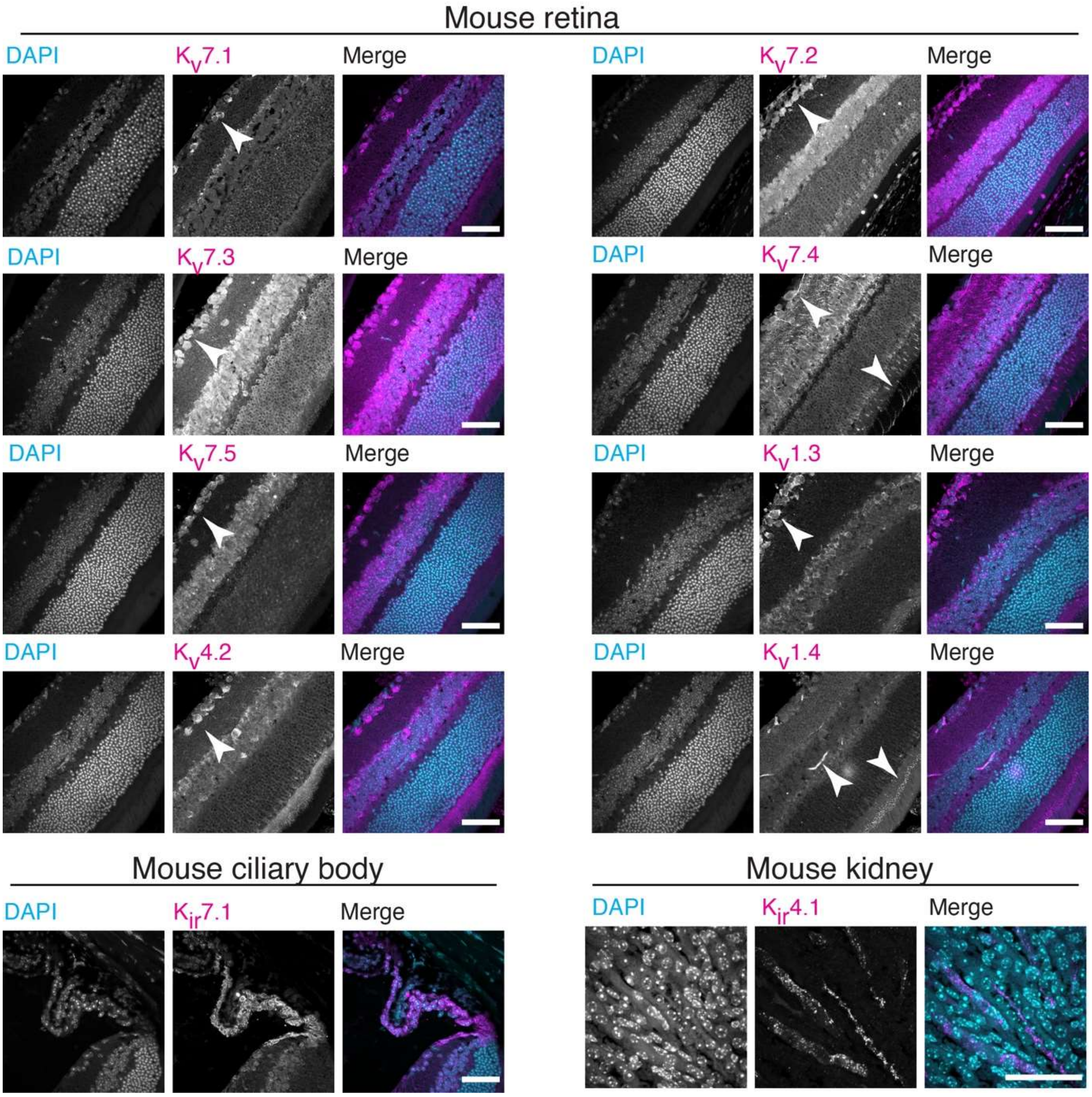

Supplementary Figure S5. Specificity of K^+ channel antibodies. Immunostainings of mouse paraffin-embedded tissue sections represented as xy-maximum intensity projections of confocal images with K^+ channels shown in magenta and cell nuclei (DAPI) in blue. Channels K_V7.1-7.5 are expressed in the retina, as previously reported [63], localizing especially to ganglion cells (K_V7.1-7.5), and K_V7.4 also to photoreceptor inner segments. Ganglion cells are correctly immunopositive for K_V1.3 and K_V4.2, and photoreceptor inner segments and retinal vessels for K_V1.4. Immunopositivity for Kir7.1 is shown in the mouse ciliary body non pigmented epithelial cells and for Kir4.1 in mouse kidney. Scale bars 20 µm.
